# Quantum Density Peak Clustering Algorithm

**DOI:** 10.3390/e24020237

**Published:** 2022-02-03

**Authors:** Zhihao Wu, Tingting Song, Yanbing Zhang

**Affiliations:** 1 College of Cyber Security, Jinan University, Guangzhou 510632, China; zhihaowu@stu2019.jnu.edu.cn; 2 College of Information Science and Technology, Jinan University, Guangzhou 510632, China; yanbing@stu2019.jnu.edu.cn; 3 Guangxi Key Laboratory of Cryptography and Information Security, Guilin 541004, China

**Keywords:** quantum information, quantum computation, quantum algorithm

## Abstract

A widely used clustering algorithm, density peak clustering (DPC), assigns different attribute values to data points through the distance between data points, and then determines the number and range of clustering by attribute values. However, DPC is inefficient when dealing with scenes with a large amount of data, and the range of parameters is not easy to determine. To fix these problems, we propose a quantum DPC (QDPC) algorithm based on a quantum DistCalc circuit and a Grover circuit. The time complexity is reduced to $O(\log\left(N^{2}\right)+6N+\sqrt{N})$, whereas that of the traditional algorithm is $O(N^{2})$. The space complexity is also decreased from O(N·⌈logN⌉) to O(⌈logN⌉).

## 1. Introduction

Cluster analysis originated from taxonomy, as an ancient skill mastered by human beings. In the past, people used to classify goods based on their experience and professional knowledge. With the development of modern society, people have higher and higher requirements for classification [1,2]. Classification based only on experience and professional knowledge has been gradually eliminated, and computer technology is now used for cluster analysis, using algorithms to address huge and complex cluster tasks [3,4]. Therefore, clustering algorithms have been proposed for applications in various settings [5,6]. Moreover, the world of massive data that we live in also makes the clustering process indispensable. Many research fields are faced with the problem of a large amount of data [7,8]. If there is no preprocessing such as clustering or data dimension reduction, it is difficult to carry out subsequent analysis [9,10,11]. For example, in the area of machine learning, the original entry of almost all important algorithms is a large amount of large-scale data. It is difficult to use these data without clustering or a dimensionality reduction [12,13,14]. In the field of quantum communication, quantum communication equipment is only supplied to few large companies. Many parties in quantum communication may be classical. Clustering algorithms can help communication parties to deal with the transmitted information more conveniently [15,16,17]. In the area of data dimension reduction, we are familiar with the principal component analysis algorithm (PCA) [18], multidimensional scaling (MDS), linear discrimination (LDA), locally linear embedding (LLE), and so on [19,20,21,22]. However, the dimension reduction algorithm will inevitably reduce the attribute value of the data. If the operation is improper, the data will lose accuracy and the results will have deviated. A clustering algorithm can be used to avoid such problems. Nowadays, clustering algorithms can be divided in the following way.

Partition-based clustering algorithms include K-means [23], K-medians [24], and kernel K-means algorithms [25]. Hierarchy-based clustering algorithms include BIRCH, CURE, and the CHAMELEON algorithm [26]. Density-based clustering algorithms include DBSCAN, mean-shift (MS) [27], and the density peak clustering algorithm (DPC) [28]. Each clustering algorithm has its own advantages and disadvantages, and each algorithm has its own suitable scenarios [29]. The advantage of the DPC algorithm is that there is no need to define the number of clusters, as in the K-means algorithm. Secondly, it can detect non-spherical data, which has high application value in computer image processing. In addition, it can automatically identify abnormal points, which is also a prominent advantage of many clustering algorithms.

In 2014, Rodriguez and Laio proposed a DPC algorithm, which can automatically find the cluster center and achieve efficient clustering of arbitrarily shaped data sets [30]. DPC is a clustering algorithm based on density, and its input parameters are less than those of the K-means algorithm [31,32] and the K-medians algorithm [33,34]. The process of DPC clustering does not need to map data to vector space, which reduces the computational complexity of the algorithm.

However, the DPC algorithm still has its drawbacks. When it deals with large amounts of data, the speed of the algorithm is significantly reduced. The algorithm computes the distance between the current data point and each data point in the set, so the complexity of this operation is $O(N^{2})$, whereas *N* represents the number of data points [35]. At the same time, the process of the algorithm stores the distance between each data point and its remaining data points, which requires a large amount of storage space.

Some years ago, quantum technology was introduced to speed up the classical algorithms with large data volume, such as the Internet of Things industry and computer vision [36,37,38]. Typical quantum algorithms include the quantum K-means algorithm [32] and quantum principal component analysis [18]. They are not simple quantum version of classical algorithms. The running speeds of these quantum algorithms are greatly reduced. In this paper, we propose a QDPC algorithm, which applies a quantum DistCalc circuit to speed up the DPC algorithm.

In Section 2, the principle and flow of the classical DPC algorithm are introduced in detail. In Section 3, we propose the QDPC algorithm and its corresponding quantum circuit. In Section 4, the simulation experiments are discussed. An analysis of complexity and our conclusions are presented in Section 5.

## 2. Preliminary

### 2.1. Notation and Definitions

DPC is an algorithm that does not require iteration and can find the clustering center in one run. Distance information is the most important form of information that one must collect in the DPC algorithm. Based on the distance, one can compute the local density value.

The main ideas of DPC are based on the following assumptions:

* The clustering center has a relatively high local density value and is surrounded by data points with a low local density value.
* The clustering center is far away from any point with a higher local density value.

For each data point $x_{i}$, the algorithm computes two attribute values of the data point: its local density $\rho_{i}$ and its distance $\delta_{i}$ from the nearest higher density point. Both attribute values depend only on the distance $d_{ij}$ between the current data point $x_{i}$ and the rest of the data points $x_{j}$.

The local density $\rho_{i}$ of data point $x_{i}$ is defined as

(1) $$\rho_{i}=\sum_{j}\chi \left(d_{ij}-d_{c}\right),$$

where $d_{ij}$ is the distance between data point $x_{i}$ and $x_{j}$, $d_{c}$ is the cutoff distance. The function χ is defined as

(2) $$\chi \left(x\right)=\left\{\begin{array}{cc} 1 & \;x<0\\ 0 & \;otherwise, \end{array}\right.$$

which indicates the number of data points with distance from the data point $x_{i}$ less than the cutoff distance.

The distance from the higher density point of data point $x_{i}$ is defined as

(3) $$\delta_{i}=\min_{j,\rho_{i}<\rho_{j}}\left(d_{ij}\right),$$

where $\delta_{i}$ records the nearest distance from data point $x_{i}$ to all data points with higher local density. If $\delta_{i}$ is very small, there is a data point $x_{j}$ with a higher local density around this data point $x_{i}$. As for the data point with the highest local density, it cannot find a data point with a higher local density, and its distance is $\delta_{i}=\max_{j}\left(d_{ij}\right)$ conventionally. It can be found that when the distance $\delta_{i}$ is relatively large, this data point is the clustering center.

Therefore, after the two attribute values $\rho_{i}$ and $\delta_{i}$ of each data point are obtained, these data points are divided according to the rules.

- If the value sof $\rho_{i}$ and $\delta_{i}$ are both anomalously large, it is the clustering center;
- If the value of $\rho_{i}$ is relatively large and $\delta_{i}$ is relatively small, it is the point in a cluster;
- If the value of $\rho_{i}$ is relatively small and $\delta_{i}$ is relatively large, it is an outlier.

According to the above rules, the algorithm can accurately find every clustering center of the cluster and cluster each data point.

### 2.2. The Workflow of the Classical DPC Algorithm

The main processes of the algorithm consist of calculating two attribute $\rho_{i}$ and $\delta_{i}$ values of each data point. Suppose we have a data set with large amounts of data points $D=\{x_{1},x_{2},x_{3},\cdots ,x_{N}\}$, and the dimension of each data point is *d*. The steps of the DPC algorithm are as follows:

a. Calculate the local density $\rho_{i}$ of each data point $x_{i}$.
b. For each data point $x_{i}$, the nearest distance of $x_{i}$ is found in all data points with higher local density than $x_{i}$, and record this distance as $\delta_{i}$.
c. According to $\rho_{i}$ and $\delta_{i}$ of each data point to determine the clustering center. If $\rho_{i}$ and $\delta_{i}$ of a data point are relatively large, it is the clustering center.
d. Assign each data point to the nearest clustering center.

It should be noted that if $\delta_{i}$ of a data point is large and $\rho_{i}$ is small, then the point is an exception. It does not need to be assigned to any cluster.

## 3. QDPC Algorithm

The classical algorithm takes the largest proportion of time to calculate the distance in the whole algorithm, so quantum circuits are used to optimize this part [39,40]. In quantum technology, fidelity is an important concept, which is similar to cosine similarity [41,42] in the classical framework. Fidelity can measure the similarity between two quantum states. If the value of fidelity is 1, the two quantum states are the same; if the value of fidelity is 0, the two quantum states are orthogonal. Therefore, the distance between data points can be calculated via fidelity only if the classical data are encoded into a quantum state.

**Figure 1:**
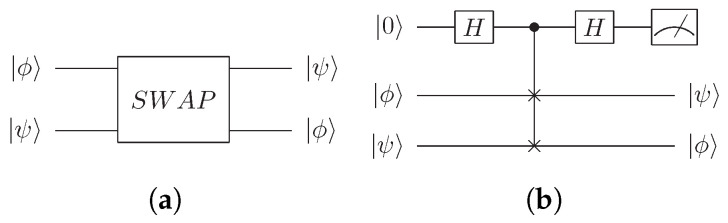
(**a**) Quantum SwapTest circuit to obtain the similarity between two quantum states |ϕ〉 and |ψ〉. (**b**) Details of quantum SwapTest circuit.

The most commonly used quantum circuit to achieve fidelity is the SwapTest. This quantum circuit was proposed by Aïmeur et al in [43]. By taking the inner product of two quantum states |ϕ〉 and |ψ〉, the SwapTest circuit is used to calculate the fidelity of quantum states, as shown in Figure 1.

**Figure 2:**
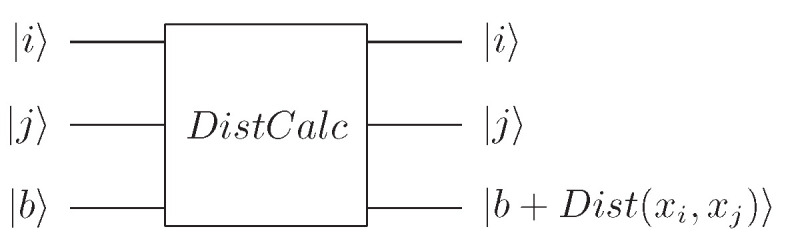
Quantum DistCalc circuit for calculating the distance between $x_{i}$ and $x_{j}$.

Based on the SwapTest circuit, the quantum DistCalc circuit [44] in Figure 2 can calculate the distance between data points $x_{i}$ and $x_{j}$. The distance is stored in the third register.

### Procedure of the QDPC Algorithm

Consider a set with *N* data points $D=\{x_{1},x_{2},x_{3},\cdots ,x_{N}\}$. The dimension of each data point is *d*. Regardless of the number of clusters, QDPC will calculate two attribute values $\rho_{i}$ and $\delta_{i}$ for each data point. Then the clustering center is determined using these two attribute values.

**Figure 3:**
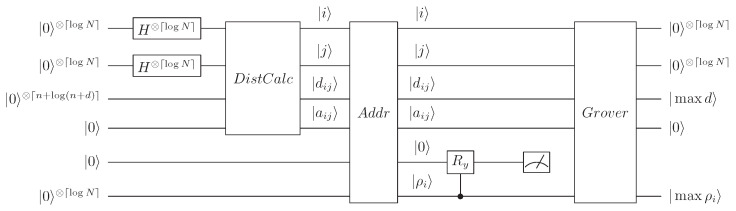
Overview of the quantum circuit for the QDPC algorithm, where $|d_{ij}\rangle$ represents $|d(x_{i},x_{j})\rangle$.

An overview of the circuit for solving the QDPC is shown in Figure 3.

The procedure used to cluster $x_{i}$ includes the following seven steps:

(i). Prepare six registers in ${|0\rangle }^{⊗\lceil \log N\rceil }⊗{|0\rangle }^{⊗\lceil \log N\rceil }⊗{|0\rangle }^{⊗\lceil n+\log(n+d)\rceil }⊗|0\rangle ⊗|0\rangle ⊗|0\rangle {}^{⊗\lceil \log N\rceil }$, and apply an *H* gate on each qubit in the first and second registers. The third register records the quantum state of the distance from two data points $x_{i}$ and $x_{j}$. The fourth register stores the intermediate conversion value $a_{ij}$, which will be explained in more detail later. The fifth register is an ancillary qubit. The last register, the sixth register, records the attribute value $\rho_{i}$. By means of quantum DistCalc, the system state is
  (4) $$|\Psi_{0}\rangle =\frac{1}{N}\sum_{i=1}^{N}\sum_{j=1}^{N}\left(|i\rangle ⊗|j\rangle ⊗|d\left(x_{i},x_{j}\right)\rangle \right)⊗|0\rangle ⊗|0\rangle ⊗{|0\rangle }^{⊗\lceil \log N\rceil }+|G\rangle ,$$
  where |G〉 is a garbage state.
(ii). Set a desired threshold as $d_{max}$ and set $a_{ij}\in \left\{0,1\right\}$ to indicate whether two data points $x_{i}$ and $x_{j}$ are close together. The value of $a_{ij}$ is 1 if the distance of two data points $d\left(x_{i},x_{j}\right)\leq d_{max}$, otherwise 0. Then we can easily store this value $a_{ij}$ in the fourth register. The system state is
  (5) $$|\Psi_{1}\rangle =\frac{1}{N}\sum_{i=1}^{N}\sum_{j=1}^{N}\left(|i\rangle ⊗|j\rangle ⊗|d\left(x_{i},x_{j}\right)\rangle ⊗|a_{ij}\rangle \right)⊗|0\rangle ⊗{|0\rangle }^{⊗\lceil \log N\rceil }+|G\rangle .$$
(iii). Take a control-sum operation on the first, fourth, and sixth register. The first register |i〉 is the control qubit, and the sixth register stores the sum of the values of $a_{ij}$, whereas the index *i* is fixed. Since the local density property of the data point $x_{i}$ is $\rho_{i}=\sum_{j=1}^{N}a_{ij}$, the value stored in the sixth register is $|\rho_{i}\rangle$. Now the system is
  (6) $$|\Psi_{2}\rangle =\frac{1}{N}\sum_{i=1}^{N}\left[|i\rangle \sum_{j=1}^{N}\left(|j\rangle ⊗|d\left(x_{i},x_{j}\right)\rangle ⊗|a_{ij}\rangle \right)⊗|0\rangle ⊗|\rho_{i}\rangle \right]+|G\rangle .$$
(iv). Perform a control conditional rotation [45], where the first and the second registers are control qubits, and the fifth ancillary register is the target. Set the fifth register to |1〉, when $\rho_{j}>\rho_{i}$, otherwise set to |0〉. The whole system is divided into two parts, as shown below
  (7) $$\begin{array}{ccc} & & |\Psi_{3}\rangle =\frac{1}{N}\sum_{i=1}^{N}\left[|i\rangle \left(\sum_{j,\rho_{j}>\rho_{i}}|j\rangle |d\left(x_{i},x_{j}\right)\rangle |a_{ij}\rangle |1\rangle +\sum_{j,\rho_{j}\leq \rho_{i}}|j\rangle |d\left(x_{i},x_{j}\right)\rangle |a_{ij}\rangle |0\rangle \right)⊗|\rho_{i}\rangle \right]+|G\rangle . \end{array}$$
(v). Apply a projection operation {|0〉〈0|,|1〉〈1|} on the fifth register, and keep the state when the measurement result is |1〉〈1|. The system is
  (8) $$|\Psi_{4}\rangle =\Gamma |\Psi_{3}\rangle =\alpha \sum_{i=1}^{N}\sum_{j,\rho_{i}<\rho_{j}}^{N}|i\rangle |j\rangle |d\left(x_{i},x_{j}\right)\rangle |a_{ij}\rangle |1\rangle |\rho_{i}\rangle +|G\rangle ,$$
  where α is the normalized parameter, and ${\sum |\alpha |}^{2}=1$. The third register and the last register store the attribute values $\delta_{i}$ and $\rho_{i}$ of each data point $x_{i}$, respectively.
(vi). Perform a bit flip operation [46] on the third register, and the value is changed from $d(x_{i},x_{j})$ to $\bar{d(x_{i},x_{j})}$. By doing this, the minimum value in the third register becomes the maximum value. In order to make the following Grover algorithm run under more convenient conditions, we change the target of the search to the maximum value of the two attributes. Data points that meet these two requirements are the center of clustering. Now the system is
  (9) $$|\Psi_{5}\rangle =\alpha \sum_{i=1}^{N}\sum_{j,\rho_{i}<\rho_{j}}^{N}\left|i\rangle |j\rangle \right|\bar{d(x_{i},x_{j})}\rangle |a_{ij}\rangle |1\rangle |\rho_{i}\rangle +|G\rangle .$$
(vii). Apply the Grover algorithm [47] to find the index *i* of data point $x_{i}$ with maximum $\rho_{i}$ and the index *j* of the found data point $x_{i}$ with maximum $\delta_{i}$ with a full successful probability. The index *i* that meets the requirements is the center of a cluster.

## 4. Simulation Results

We clustered three differently distributed (horizontally, circularly, and discrete) data sets using our QDPC algorithm, implemented on Baidu’s quantum platform Paddle Quantum. Limited to the lack of QRAM devices, thread concurrency was used to read out all the data in a data set at one time. Data were generated by a random function with seed=21, and the number of data *N* was fixed as 20,40,80,250,500, and 1000. Table 1 gives the common evaluation indicators purity, F-score and adjusted Rand index (ARI) of the two algorithms on the circularly distributed data. In the table, all the values lie between 0.95 and 1. When N=20,40,80, the clustering results of the DPC algorithm are the same as those of the QDPC algorithm. For N=20,40,80, the values of QDPC are greater than those of DPC, so the QDPC performs better than the DPC.

**Figure 4:**
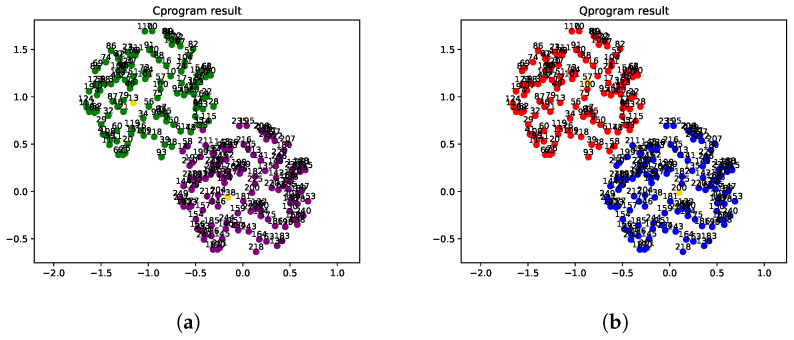
Experimental results of two algorithms when the data are circularly distributed and the number *N* is fixed as 250. (**a**) Clustering performance of DPC algorithm; (**b**) clustering performance of QDPC algorithm.

We also depict the clustering performance of two algorithms when *N* is fixed at 250 in Figure 4. All the points are accompanied with their indexes. The points colored yellow are the centers of the clusters. Other points colored the same are clustering together, so both DPC and QPDC cluster the data into two groups. But DPC performs slightly worse than QDPC, since the points with indexes 14, 58 and 33 are colored with green, which should be purple.

The experiment was repeated 10 times and the average running times are recorded in Table 2. It can be seen that with increasing *N*, the running time of QDPC increases linearly, and that of the DPC increases exponentially. When *N* is fixed at 250,500, or 1000, QDPC is faster than DPC, but when *N* is less than 80, DPC is faster than QDPC. The reason for this may be the fact that we simulated these results on a classical computer. If we ran the QDPC algorithm on a real quantum computer, the results may show an improvement.

## 5. Discussion and Conclusions

We now analyze the complexity of the QDPC algorithm step by step. In (i), a quantum DistCalc circuit is applied to obtain the distance between two data points. The time complexity of this step depending on the distance definition is log(N·N) [44]. In (ii) and (iii), we convert the value $d(x_{i},x_{j})$ into $a_{ij}$ and add up $a_{ij}$. The time complexity of these two steps can be measured by the number of register accesses and the quantum addition circuit. Therefore, in general, the time complexity of this part is O(N+5N) [48]. In (iv), (v), and (vi), we perform the conditional rotation operation, the projection operation, and the bit flip operation. The time complexity is relatively negligible compared with other steps. Finally, step (vii) requires the application of the Grover algorithm, which introduces a time complexity of $O(\sqrt{N})$ [49,50]. Thus, the time complexity of the whole algorithm is $O(\log\left(N^{2}\right)+6N+\sqrt{N})$. The space complexity is the space size of the quantum registers, i.e., ⌈logN⌉+⌈logN⌉+⌈n+log(n+d)⌉+1+1+⌈logN⌉=3⌈logN⌉+⌈n+log(n+d)⌉+2.

For the DPC algorithm, the most time-consuming step is to calculate the distance between data points. It can be seen that the total distances of $\frac{1}{2}N\left(N-1\right)$ times need to be calculated [51]. So the complexity of the classical DPC algorithm is $O(N^{2})$. The space complexity of DPC depends on the space stored, $\rho_{i}$ and $\delta_{i}$, for each point. The space required is N·⌈logN⌉+N·⌈n+log(n+d)⌉ bits.

A corresponding comparison between classical and quantum algorithms is shown in Table 3. Based on Table 3, the QDPC algorithm costs less than the DPC algorithm in terms of both time and space complexities.

In this paper, we have proposed a QDPC algorithm that is more efficient in both time and space than the classical algorithm. We applied it to two key circuits, a quantum DistCalc circuit and a Grover circuit. The quantum DistCalc circuit calculates the distance between data points in the data set, from which two important attribute values, $\rho_{i}$ and $\delta_{i}$, required by the QDPC algorithm are obtained. Then, the Grover algorithm is used to search the index of clustering center points that meet the conditions from the data set. In the future, we will investigate some possible application scenarios of the QDPC algorithm and compare the efficiency of algorithms on different data set structures.

## Figures and Tables

**Table 1:** Evaluation indicators of circularly distributed data.

*Seed* = 21	DPC			QDPC		
	Purity	F-Score	ARI	Purity	F-Score	ARI
*N* = 20	1.0	1.0	1.0	1.0	1.0	1.0
*N* = 40	1.0	1.0	1.0	1.0	1.0	1.0
*N* = 80	1.0	1.0	1.0	1.0	1.0	1.0
*N* = 250	0.988000	0.976104	0.952385	1.0	1.0	1.0
*N* = 500	0.992000	0.984066	0.968192	0.996000	0.992000	0.984032
*N* = 1000	0.990000	0.980164	0.960360	0.998000	0.996000	0.992008

**Table 2:** Comparison of the complexity of the two algorithms in simulation experiments.

Algorithms	N=20	N=40	N=80	N=250	N=500	N=1000
DPC(/s)	0.00500488	0.01701570	0.06606030	0.65659642	2.54531145	10.08315921
QDPC(/s)	0.02302074	0.03603339	0.06806207	0.19617867	0.39135551	0.77470422
Difference(/s)	−0.01801586	−0.0190176	−0.00200177	0.46041775	2.15395594	9.30845499

**Table 3:** Theoretical comparison of the complexity of the two algorithms.

Complexity	DPC	QDPC
time	$O(N^{2})$	$O(\log\left(N^{2}\right)+6N+\sqrt{N})$
space	N·⌈logN⌉+N·⌈n+log(n+d)⌉	3⌈logN⌉+⌈n+log(n+d)⌉+2

## Data Availability

Not applicable.
